# Supplementary material for: Dopaminergic action prediction errors serve as a value-free teaching signal
Source: Nature. 2025 May 14;643(8074):1333–42. doi: 10.1038/s41586-025-09008-9 (PMC12310545; doi:10.1038/s41586-025-09008-9)
Supplement: Supplementary file 1 — Reporting Summary [file 41586_2025_9008_MOESM1_ESM.pdf]

nature portfolio

Corresponding author(s): Marcus Stephenson-Jones

Last updated by author(s): Mar 28, 2025

Reporting Summary

Nature Portfolio wishes to improve the reproducibility of the work that we publish. This form provides structure for consistency and transparency in reporting. For further information on Nature Portfolio policies, see our Editorial Policies and the Editorial Policy Checklist.

Statistics

For all statistical analyses, confirm that the following items are present in the figure legend, table legend, main text, or Methods section.

n/a	Confirmed	
☐	☒	The exact sample size (*n*) for each experimental group/condition, given as a discrete number and unit of measurement
☐	☒	A statement on whether measurements were taken from distinct samples or whether the same sample was measured repeatedly
☐	☒	The statistical test(s) used AND whether they are one- or two-sided<br>*Only common tests should be described solely by name; describe more complex techniques in the Methods section.*
☐	☒	A description of all covariates tested
☐	☒	A description of any assumptions or corrections, such as tests of normality and adjustment for multiple comparisons
☐	☒	A full description of the statistical parameters including central tendency (e.g. means) or other basic estimates (e.g. regression coefficient) AND variation (e.g. standard deviation) or associated estimates of uncertainty (e.g. confidence intervals)
☐	☒	For null hypothesis testing, the test statistic (e.g. *F*, *t*, *r*) with confidence intervals, effect sizes, degrees of freedom and *P* value noted<br>*Give P values as exact values whenever suitable.*
☒	☐	For Bayesian analysis, information on the choice of priors and Markov chain Monte Carlo settings
☒	☐	For hierarchical and complex designs, identification of the appropriate level for tests and full reporting of outcomes
☐	☒	Estimates of effect sizes (e.g. Cohen's *d*, Pearson's *r*), indicating how they were calculated

Our web collection on statistics for biologists contains articles on many of the points above.

Software and code

Policy information about availability of computer code

Data collection

Custom Matlab and python code was used to run experimental paradigms.

Data analysis

Custom Matlab and python code was used for data analysis. The analysis code is available at https://doi.org/10.5281/zenodo.15103777. For analysis the following version numbers were used

Photometry data analysis: Python 3.8, numpy==1.21.5, matplotlib==3.5.1, seaborn==0.11.2, scipy==1.7.3 tqdm==4.63.0, pandas==1.4.1, scikit-learn==1.0.2, cycler==0.11.0, scikit-image==0.19.3, nptdms==1.6.2, peakutils==1.3.3, statsmodels==0.13.5, cmocean==4.0.3, openpyxl==3.0.9.

Modeling: Python 3.7, numpy==1.19.0, pandas==1.0.5, matplotlib==3.2.2, tqdm==4.50.0, statsmodels==0.13.5

Behavioural and manipulation data analysis: matplotlib==3.5.2, numpy==1.23.1, pandas==1.4.3, scikit-learn==1.1.2, scipy==1.9.0, seaborn==0.11.2

For manuscripts utilizing custom algorithms or software that are central to the research but not yet described in published literature, software must be made available to editors and reviewers. We strongly encourage code deposition in a community repository (e.g. GitHub). See the Nature Portfolio guidelines for submitting code & software for further information.

Data

Policy information about availability of data

All manuscripts must include a data availability statement. This statement should provide the following information, where applicable:

- Accession codes, unique identifiers, or web links for publicly available datasets
- A description of any restrictions on data availability
- For clinical datasets or third party data, please ensure that the statement adheres to our policy

All of the datasets needed to reproduce the findings in the paper are available at, https://rdr.ucl.ac.uk/projects/Dopaminergic_action_prediction_errors_serve_as_a_value-free_teaching_signal/240596.

In addition Allen Brain Atlas connectivity data was used to demarcate the cortico-striatal projection patterns from AUDp (experiments, 100149109, 116903230, 120491896 and 146858006) and S1(SSp) (experiments 112882565, 114290938 and 126908007).

Research involving human participants, their data, or biological material

Policy information about studies with human participants or human data. See also policy information about sex, gender (identity/presentation), and sexual orientation and race, ethnicity and racism.

Reporting on sex and gender	N/A
Reporting on race, ethnicity, or other socially relevant groupings	N/A
Population characteristics	N/A
Recruitment	N/A
Ethics oversight	N/A

Note that full information on the approval of the study protocol must also be provided in the manuscript.

Field-specific reporting

Please select the one below that is the best fit for your research. If you are not sure, read the appropriate sections before making your selection.

☒ Life sciences ☐ Behavioural & social sciences ☐ Ecological, evolutionary & environmental sciences

For a reference copy of the document with all sections, see nature.com/documents/nr-reporting-summary-flat.pdf

Life sciences study design

All studies must disclose on these points even when the disclosure is negative.

Sample size	Sample size calculations were not performed but preliminary experiments were performed to gauge variation and help guide the choice of sample size.
Data exclusions	One experimental animal was excluded from the dopamine cell ablation experiment as the lesion quantification showed no ablation (this mouse performance was comparable to controls). Mice that were targeted for TS recordings were excluded if serial two-photon microscopy confirmed that the fibers were located outside of the TS (n=8).
Replication	None of the findings were explicity replicated.
Randomization	Cage mates were assigned to experimental and control groups. This was done without explicit randomization procedures.
Blinding	Experimenters were not blind to the whether mice were in the control or manipulation groups.

Reporting for specific materials, systems and methods

We require information from authors about some types of materials, experimental systems and methods used in many studies. Here, indicate whether each material, system or method listed is relevant to your study. If you are not sure if a list item applies to your research, read the appropriate section before selecting a response.

Materials & experimental systems

n/a	Involved in the study	
☐	☒	Antibodies
☒	☐	Eukaryotic cell lines
☒	☐	Palaeontology and archaeology
☐	☒	Animals and other organisms
☒	☐	Clinical data
☒	☐	Dual use research of concern
☒	☐	Plants

Methods

n/a	Involved in the study	
☒	☐	ChIP-seq
☒	☐	Flow cytometry
☒	☐	MRI-based neuroimaging

Antibodies

Antibodies used	NeuN 1:1000 (abcam, ab104225), tyrosine hydroxylase (TH) 1:1000 (Sigma-Aldrich, AB152), GFP 1:1000 (Aves labs, GFP-1020), Alexa-488 anti-mouse 1:500 (Invitrogen, AB_2534069), Alexa-567 anti-chicken 1:500 (Invitrogen, AB_2535858), and Alexa-647 anti-rabbit 1:500 (Invitrogen, AB_2535813).
Validation	ab104225: According to the manufacturer antibody specificity was analyzed by western blot analysis, where it specifically detects NeuN (UniProt ID: A6NFN3; Molecular weight: 34kDa). It has been cited in over 245 publications.<br>AB152: According to the manufacturer the antibody selectively labels in a western blot analysis a single band at approximately 62kDa (reduced) corresponding to Tyrosine Hydroxylase.It has been cited in over 100 publications.<br>GFP-1020: cited in 2347 publications and tested according to the manufacturer with western blot analysis.

Animals and other research organisms

Policy information about studies involving animals; ARRIVE guidelines recommended for reporting animal research, and Sex and Gender in Research

Laboratory animals	Male and female adult mice between the ages of 2-7 months from the following mouse lines were used: C57BL/6J wild-type (Charles River), Drd1-Cre (Gensat: EY262), Adora2a-Cre (Gensat: KG139), DAT-Cre (JAX Stock No: 006660), Ai14 (tdTomato, JAX Stock No: 007914), Ai35 (Arch-GFP, JAX Stock No: 012735) and Ai32 (channelrhodopsin-2/EYFP, JAX Stock No: 024109)
Wild animals	N/A
Reporting on sex	Male and female adult mice were used in all experiments
Field-collected samples	N/A
Ethics oversight	All experiments were performed in accordance with the UK Home Office regulations Animal (Scientific Procedures) Act 1986 and the Animal Welfare and Ethical Review Body (AWERB).

Note that full information on the approval of the study protocol must also be provided in the manuscript.

Plants

Seed stocks	*Report on the source of all seed stocks or other plant material used. If applicable, state the seed stock centre and catalogue number. If plant specimens were collected from the field, describe the collection location, date and sampling procedures.*
Novel plant genotypes	*Describe the methods by which all novel plant genotypes were produced. This includes those generated by transgenic approaches, gene editing, chemical/radiation-based mutagenesis and hybridization. For transgenic lines, describe the transformation method, the number of independent lines analyzed and the generation upon which experiments were performed. For gene-edited lines, describe the editor used, the endogenous sequence targeted for editing, the targeting guide RNA sequence (if applicable) and how the editor was applied.*
Authentication	*Describe any authentication procedures for each seed stock used or novel genotype generated. Describe any experiments used to assess the effect of a mutation and, where applicable, how potential secondary effects (e.g. second site T-DNA insertions, mosiacism, off-target gene editing) were examined.*
